# Identification of Differentially Expressed Genes between Original Breast Cancer and Xenograft Using Machine Learning Algorithms

**DOI:** 10.3390/genes9030155

**Published:** 2018-03-12

**Authors:** Deling Wang, Jia-Rui Li, Yu-Hang Zhang, Lei Chen, Tao Huang, Yu-Dong Cai

**Affiliations:** 1 Institute of Health Sciences, Shanghai Institutes for Biological Sciences, Chinese Academy of Sciences, Shanghai 200031, China; wangdl@sysucc.org.cn (D.W.); zhangyh825@163.com (Y.-H.Z.); 2 Department of Medical Imaging, Sun Yat-sen University Cancer Center, State Key Laboratory of Oncology in South China; Collaborative Innovation Center for Cancer Medicine, Guangzhou 510060, China; 3 School of Life Sciences, Shanghai University, Shanghai 200444, China; jiaruili@shu.edu.cn; 4 College of Information Engineering, Shanghai Maritime University, Shanghai 201306, China; chen_lei1@163.com

**Keywords:** Monte Carlo feature selection, breast cancer, random forest, patient-derived tumor xenograft

## Abstract

Breast cancer is one of the most common malignancies in women. Patient-derived tumor xenograft (PDX) model is a cutting-edge approach for drug research on breast cancer. However, PDX still exhibits differences from original human tumors, thereby challenging the molecular understanding of tumorigenesis. In particular, gene expression changes after tissues are transplanted from human to mouse model. In this study, we propose a novel computational method by incorporating several machine learning algorithms, including Monte Carlo feature selection (MCFS), random forest (RF), and rough set-based rule learning, to identify genes with significant expression differences between PDX and original human tumors. First, 831 breast tumors, including 657 PDX and 174 human tumors, were collected. Based on MCFS and RF, 32 genes were then identified to be informative for the prediction of PDX and human tumors and can be used to construct a prediction model. The prediction model exhibits a Matthews coefficient correlation value of 0.777. Seven interpretable interactions within the informative gene were detected based on the rough set-based rule learning. Furthermore, the seven interpretable interactions can be well supported by previous experimental studies. Our study not only presents a method for identifying informative genes with differential expression but also provides insights into the mechanism through which gene expression changes after being transplanted from human tumor into mouse model. This work would be helpful for research and drug development for breast cancer.

## 1. Introduction

According to epidemiological data from the World Health Organization (WHO), breast cancer has been confirmed to be the most frequently diagnosed cancer in women from developed and developing countries and is accompanied with rising expectancy of average life span [1,2]. Early in 2011, more than 508,000 women died from breast cancer, accounting for more than 58% in developing countries [1]. However, the incidence of breast cancer is higher in Western Europe (89.7/100,000) is higher than those in Eastern Africa (19.3/100,000) and most developing countries (40/100,000); this finding reflects the specific regional distribution of breast cancer incidence [3] and suggests the need for development of precise and effective methods for diagnosis and treatments.

To identify a cure for breast cancer, researchers have applied five levels of research, namely, molecular, cellular, histopathological, animal model, and clinical levels [4]. Among these levels, the animal model level not only confirms the conclusion obtained from studies on the other levels as an integrated creature but also lays the foundation for further clinical studies [5,6]. In studies on this level, mice are a typical and widely used species because of their high degree of genetic similarity to human beings and simple cultivation requirements. However, murine tumors considerably differ from human tumors at both genetic and molecular levels [7]. Therefore, a murine tumor model cannot thoroughly reflect the characteristics of human tumors. To solve this limitation, scholars have developed patient-derived tumor xenograft (PDX) model with various modified subtypes. In general, PDX mouse model is produced by directly transplanting cancerous tissues from a unique patient into an immune-deficient mouse model; this model can be used for studies on the biological, pharmacological, or clinical characteristics of such tumor tissues [8,9]. In comparison with conventional xenograft models using cell lines, the PDX model retains the molecular signatures and cancerous heterogeneity, which includes the cancer stem cells of the original tumor [10].

Although the PDX mouse model maintains most of the characteristics of the original tumor, genomic and transcriptional variations induced by murine microenvironment selection have been characterized. In 2014, researchers from University of North Carolina (USA) found that in pancreatic and colorectal cancer, the mutation frequencies of two oncogenic genes, namely, *KRAS* and *PIK3CA*, were greatly altered by the murine microenvironment across passages; hence, at the genomic level, the PDX mouse model cannot completely maintain the characteristics of in situ tumor tissues [11]. At the transcriptomic level, a study on PDX tumor expression patterns of 58 patients with eight different tumors reported that 48 genes were differentially expressed between the PDX mouse model and the tumor tissues of the patients [12].

In this study, we obtained and used the gene expression data from a recent study [13] on PDX mouse models and original breast cancer to identify differentially expressed genes, which are called informative genes. We collected a total of 657 PDX and 174 human tumors. By using Monte Carlo feature selection (MCFS), we evaluated the relevance of the gene expression in distinguishing the original breast cancer and the PDX models. Subsequently, we compared several algorithms for building classifiers including random forest (RF), rough set-based rule learning, support vector machine (SVM) and dagging. We found that building classifier using RF algorithm with the 32 identified informative genes could achieve the best performance, obtaining the highest Matthews coefficient correlation (MCC) value of 0.777. This result demonstrated the predictive power of the 32 identified informative genes in distinguishing PDX tumors from original human tumors. Furthermore, we applied rough set-based rule learning method to detect human-interpretable rules to reveal the interactions between the 32 informative genes. These genes rules, and interactions were found to be supported by previous studies through a literature review. 

## 2. Materials and Methods 

Our entire data set contained 831 tumor cells, including 657 PDX and 174 tumor cells. The 831 tumor cells were encoded into feature vectors by the expression levels of 69 genes. The 69 genes we used were derived from the work by Lawson et al. [13]. To investigate metastatic breast cancer, Lawson et al. did a literature survey and collected a small set of genes that were highly possible as the key genes in breast cancer and clearly involved in breast cancer related functions, such as stemness, pluripotency, epithelial-to-mesenchymal transition (EMT), mammary lineage specification, dormancy, cell cycle and proliferation. Based on the curated gene set, they designed a special single-cell array called Biomark 96.96 dynamic array [13] which can measure the expression level of 116 genes. In our study, since some genes were not expressed in many tumor cells and did not play important roles as indicated by the literature, we only analyzed 69 genes by filtering those with missing expression in more than half of tumor cells.

Our method for identifying differentially expressed genes consisted of three steps: (i) applying MCFS method [14,15,16] to rank features; (ii) using incremental forward selection (IFS) and RF [17,18] methods to construct prediction model; and (iii) employing rough set-based rule learning [19] to detect interactions between differentially expressed genes.

### 2.1. Data Preparation and Feature Construction

The expression levels of 116 genes in 1293 tumor cells from three patients with breast cancer and the corresponding PDX models were obtained from a recent publication [13]. These genes are involved in stemness, pluripotency, epithelial-to-mesenchymal transition, mammary lineage specification, dormancy, cell cycle, and proliferation [13]; these processes suggest their crucial roles in tumor development. For the missing data in the original dataset, we filtered out those genes with missing data in more than half of tumor cells and those tumor cells with missing data in more than half of 116 genes. The rest of the missing data retained in the dataset were imputed using the nearest neighbor averaging method [20,21]; this filter process resulted to 69 genes and 831 tumor cells, in which 657 PDX mouse tumor cells and 174 original human tumor cells were found. The expression levels of the 831 tumor cells were used as features to encode each cell in this study.

### 2.2. Monte Carlo Feature Selection Method

MCFS method is designed to select informative features for supervised classifiers by randomly constructing a large number of tree classifiers from an original training dataset. MCFS assigns higher relative importance (*RI*) to a feature if this feature is selected by more tree classifiers. Suppose the total of *s×t* classification trees are constructed by selecting *s* subsets of *m* features (*m* ≪ *d*, where *d* is the total number of features), and constructing *t* trees for each of the *s* subsets. Each of the *t* trees is built and trained by a random selection of training and test sets using the original training set. The *RI* of a feature can be measured by estimating the overall number of splits involving the feature in all nodes of all trees constructed. Particularly, the *RI* of a feature *g* can be estimated by the following equation

(1) $$RI_{g}={\sum_{\tau =1}^{st}\left(wAcc\right)}^{u}\sum_{n_{g}(\tau )}IG(n_{g}(\tau )){\left(\frac{\mathrm{no}.\;\mathrm{in}\;n_{g}(\tau )}{\mathrm{no}.\;\mathrm{in}\;\tau }\right)}^{v},$$

where wAcc is the weighted accuracy over all samples, $IG(n_{g}(\tau ))$ is the information gain of the node $n_{g}(\tau )$, $\left(\mathrm{no}.\;\mathrm{in}\;n_{g}(\tau )\right)$ is the number of samples in node $n_{g}(\tau )$, (no. in τ) is the number of samples in tree τ, and u and v are fixed real numbers. 

In this study, we performed MCFS ranking by using the MCFS software package [14]. MCFS program can output a list in which features were ranked according to their *RI* values. The ranking list can be formulated as

(2) $$F=[f_{1},f_{2},\ldots ,f_{N}],$$

where *N* is the total number of features (*N* = 69 in this study). 

### 2.3. Incremental Forward Selection Method and Random Forest Classification

We used MCFS method to evaluate the importance of the 69 features, resulting in a feature list *F*. However, determining the optimal number of features to be used is difficult. Therefore, IFS [22,23,24] and RF methods [25,26] were employed to tell how many features should be used.

IFS method first constructed a series of candidate feature sets, denoted as $F_{1},F_{2},\ldots ,F_{N}$, where $F_{i}=\{f_{1},f_{2},\ldots ,f_{i}\}$ by incrementally adding features to precedent feature set according to their orders in the ranking list. For example, $F_{1}$ was composed of only the top 1 feature in the list *F*, $F_{2}$ was composed of the top 1 and top 2 features in the list *F*, and so on. To select the optimal feature set, i.e., determining the optimal features to be used, RF classification algorithm was then performed on all candidate feature sets. The classification performance of each candidate model was evaluated by cross-validation [27,28] (see Section 2.5 for details). The candidate model achieving the best performance was selected as the optimal prediction model. Candidate feature set that corresponded to the optimal model was determined as the optimal feature set. Features contained in the optimal feature set were actually the informative genes identified to be differentially expressed between PDX and human tumor cells.

### 2.4. Rough Set-Based Rule Learning

After obtaining the informative genes, the rough set-based rule learning algorithm can be applied to detect interaction between informative genes. The detected interactions were represented as rules. A rule is a terminology to describe a relation between rule conditions (the left-hand-side of the rule) and the rule outcome (the right-hand-side). For example, in our study, a rule can be presented as IF–THEN relationship based on gene expression: IF Gene1 = *high* AND Gene2 = *low* THEN type = *human cell*. Details for creating rough set-based rule model are presented below, including an introduction of rough set theory, and the repeated incremental pruning to produce error reduction (RIPPER) algorithm which was used to generate decision rules based on rough set theory.

Rough set is described briefly as follows. A decision (classification) system A can be defined as:

(3) A=(U,A∪{d}),

where U denotes all the objects, A represents all the condition attributes (features), and d is the decision attribute (class label). An attribute a∈A can be regarded as a function such that a(x) is the value of the attribute a of object x. Generalized decision attribute $\partial_{A}$ can be defined to replace d, to deal with the situation where the values of all the condition attributes of some objects are the same, but the values of the decision variable d of these objects are different [29]. A function, discerns(a,x,y,d), is defined as the situation in which object x and y are discernable using attribute a in term of the generalized decision variable $\partial_{A}$. A symmetric |U|×|U| matrix, called discernibility matrix $M_{A}^{d}$, is defined as follows

(4) $$M_{A}^{d}\left[i,j\right]=M_{A}^{d}\left(x_{i},x_{j}\right)=\left\{a\in A|discerns\left(a,x_{i},x_{j},d\right)\right\}\;i,j=1,2,\ldots ,\left|U\right|,$$

The entry $M_{A}^{d}\left(x_{i},x_{j}\right)$, i.e., the (i,j)th entry of the matrix $M_{A}^{d}$, contains all the attributes that can separate object $x_{i}$ from object $x_{j}$. For example, an entry $M_{A}^{d}\left(x_{i},x_{j}\right)=\left\{a_{1},a_{2},a_{5}\right\}$ means that $x_{i}$ and $x_{j}$ can be distinguished by $a_{1}$, $a_{2}$ or $a_{5}$.

A Boolean product-of-sums (POS) function, called discernibility function $f_{A}^{d}\left(x\right)$ that is relative to object x, can be computed from the row x of $M_{A}^{d}$ as follows

(5) $$f_{A}^{d}\left(x\right)=\prod_{y\in U}\left\{\sum a^{*}|a\in M_{A}^{d}\left(x,y\right)\;\mathrm{and}\;M_{A}^{d}\left(x,y\right)\neq \emptyset \right\},$$

where $a^{*}$ corresponds to the membership of attribute a. Function $f_{A}^{d}\left(x\right)$ is composed of some conjunctions and each conjunction contains some terms corresponding to a non-empty entry of the row x of $M_{A}^{d}$. For example, $f_{A}^{d}\left(x\right)=\left(a_{1}^{*}⋁a_{3}^{*}⋁a_{5}^{*}\right)\;⋀(a_{1}^{*}⋁a_{2}^{*})$ contains two conjunctions indicating that two entries of the row x of $M_{A}^{d}$ are not empty with one set as $\left\{a_{1},a_{3},a_{5}\right\}$ and another set as $\left\{a_{1},a_{2}\right\}$. Finding the set of all the prime implicants of $f_{A}^{d}\left(x\right)$, all the reducts, RED(A,x,d), that are relative to an object x can be determined, where a reduct is a minimal set of features that can separate x from other objects equally well with the full set of attributes A. A reduct preserves the boundaries between the approximation regions defined by the rough set [29]. A discernibility function $g_{A}^{d}\left(U\right)$ can be defined to discern all objects as follows

(6) $$g_{A}^{d}\left(U\right)=\prod_{x\in U}f_{A}^{d}\left(x\right),$$

Accordingly, the reducts RED(A,d) of the whole decision system are the prime implicants of $g_{A}^{d}\left(U\right)$. The Boolean POS function $f_{A}^{d}\left(x\right)$ and $g_{A}^{d}\left(U\right)$ can often be considerably simplified according to multiplicative idempotence and absorption [29]. However, finding all reducts using $f_{A}^{d}\left(x\right)$ or $g_{A}^{d}\left(U\right)$ is non-deterministic polynomial (NP)-hard, and for many applications only one reduct is needed. In our work, a heuristic method called Johnson Reducer algorithm [29] is applied to find a single reduct which is generally close to minimal size. 

Johnson Reducer [30], which is based on a discernibility function, first initializes the set of the reduct R(A,x,d) to be empty. Then (1) find the attribute a that appears most frequently in the remaining conjunctions of the discernibility function; (2) add attribute a to R(A,x,d); (3) remove all the conjunctions that contain a. Repeat (1), (2) and (3) until all conjunctions are removed from the discernibility function. 

**Figure 1:**
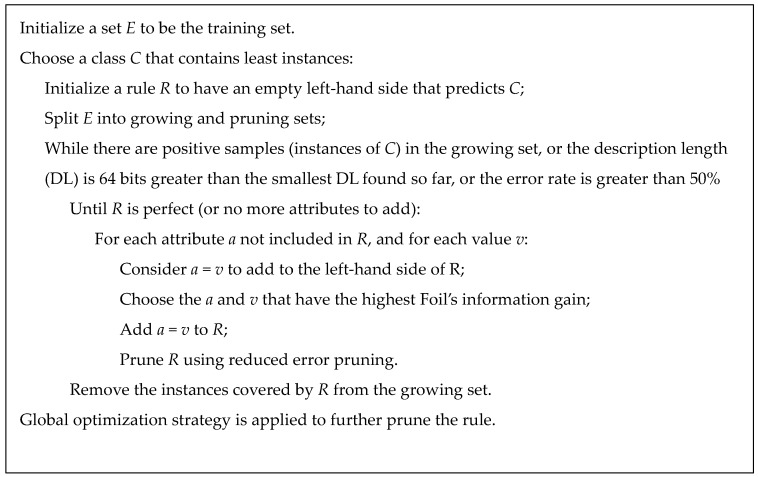
The repeated incremental pruning to produce error reduction (RIPPER) algorithm. RIPPER starts from an empty rule and splits the training set into a growing set and a pruning set. Then it repeatedly grows rules that achieve the highest Foil’s information gain using the growing set and prunes the rules using the pruning set until certain conditions are met. Finally, a global pruning is applied to prune the rules to gain the final rule set.

Based on the reduct, the RIPPER algorithm is applied to generate decision rules. RIPPER, proposed by Cohen [31] in 1995, is a rule learning algorithm which is capable of handling large noisy datasets effectively. RIPPER is the improved version of Incremental Reduced Error Pruning (IREP) [32] which combines both the separate-and-conquer technique used first in the relational learner FOIL [33], a system learning Horn clauses from data expressed as relations, and the reduced error pruning strategy proposed by Brunk and Pazzani [34]. RIPPER algorithm is described briefly in Figure 1.

In our study, we fulfilled the detection of rules by using the Johnson Reducer algorithm [29], which was also implemented in the MCFS software package [14,35].

### 2.5. Measurements

Tenfold cross-validation [27,36,37,38,39,40,41,42,43,44,45,46] was used to evaluate the prediction performance. For each fold, prediction performance was measured by the MCC. Final results were summarized as the 10-fold average. MCC considers the sample numbers among classes and is generally regarded as a balance measurement to indicate prediction accuracy even though sample numbers among classes are of very different sizes. In this study, we adopted MCC for the performance measurement because the numbers of the two classes (human cell and PDX cell) were of great imbalance (174 vs. 657).

Given the involved *n* samples, denoted by $s_{1},s_{2},\ldots ,s_{n}$, and *N* classes, represented by 1, 2, …, *N*. True classes of samples were defined as matrix $Y={\left(y_{ij}\right)}_{n\times N}$, where $y_{ij}=1$ if the $s_{i}$ belongs to class *j*; otherwise, this variable is set to 0. Predicted classes of samples were defined as matrix $X={\left(x_{ij}\right)}_{n\times N}$ $X={\left(x_{ij}\right)}_{n\times N}$, where $x_{ij}=1$, if $s_{i}$ is predicted to be class *j*; otherwise, $x_{ij}=0$. Thus, MCC is defined as

(7) $$MCC=\frac{\mathrm{cov}(X,Y)}{\sqrt{\mathrm{cov}(X,X)\mathrm{cov}(Y,Y)}},$$

where cov(*X*,*Y*) was covariance function of *X* and *Y*, which can be computed by

(8) $$\mathrm{cov}(X,Y)=\frac{1}{N}\sum_{k=1}^{N}\mathrm{cov}(x_{k},y_{k})=\frac{1}{N}\sum_{i=1}^{n}\sum_{k=1}^{N}\left(x_{ik}-{\bar{x}}_{k})(y_{ik}-{\bar{y}}_{k}\right),$$

where $x_{k}$ and $y_{k}$ denote the *k*th column of *X* and *Y*, respectively; ${\bar{x}}_{k}$ and ${\bar{y}}_{k}$ denote the mean values of numbers in $x_{k}$ and $y_{k}$, respectively. 

The range of *MCC* is between −1 and 1. With high *MCC* value, the classifier yields a good performance (1 means the given classifier yields a perfect classification, 0 indicates a classification no better than random prediction, and −1 represents a total misclassification). 

## 3. Results

**Figure 2:**
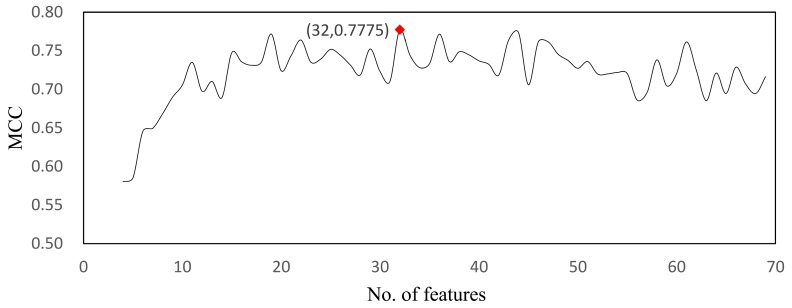
Incremental forward selection (IFS) curve illustrating the relationship of the prediction performance and the number of features incorporated in building the prediction engine. MCC: Matthews coefficient correlation.

In this study, we collected the expression levels of 69 genes in 831 tumor cells, which included 657 PDX and 167 human tumor cells. By using MCFS, we ranked the 69 genes according to their *RI* on the tumor cell classification. Then, by applying IFS and RF methods, we drew the IFS-curve, which illustrated the relationship between the number of features used for building the model and the corresponding performance measured by the MCC value (see Figure 2). As shown in Figure 2, when using the top 32 genes to build the model, the prediction performance can reach the highest MCC value of 0.777. Details of the IFS curve can be found in Supplementary Material I. 

Furthermore, we compared our method to other similar methods, including the rough-set based method as mentioned above, the SVM [47] and the dagging [48]. The comparison result was shown in Table 1. As can be seen in Table 1, using RF with the top 32 features achieved the highest MCC, the 32 genes were identified as informative genes for predicting PDX and human tumor cells. Also, we performed enrichment analysis (multiple testing corrections) on the 32 identified genes. The significant Kyoto encyclopedia of genes and genomes (KEGG) and gene ontology (GO) enrichment results of 32 genes with false discovery rate (FDR) < 0.05 can be found in Supplementary Material II.

To somewhat open the RF model, we applied the rough set-based rule learning method to detect human-interpretable IF–THEN rules to reveal the interactions between the 32 informative genes. Finally, seven IF–THEN rules were detected (see Table 2). Details of the seven IF–THEN rules were provided in Supplementary Material III, including support values, coverage, ranking of the rules, etc. Moreover, in the Section 4, comprehensive interaction analysis as well as visualization of the seven IF–THEN rules were provided.

## 4. Discussion

Based on the detailed expression profile of PDX mouse tumor tissue and the corresponding clinical tissue, we applied our newly presented computational method for the identification of core differentially expressed genes between PDX model and clinical sample. The 32 important genes used for constructing RF prediction model are listed in Table 3. All these genes have been identified to be differentially expressed in clinical tumor samples and PDX mouse model by recent publications, which validated the efficacy and accuracy of our predicted genes. Furthermore, most of these differentially expressed genes contribute to tumorigenesis; this finding implies the potential difference of oncogenic mechanisms in PDX mouse model from in situ. This study at the transcriptomic level may not only deepen our understanding on the tumorigenesis in PDX tumor mouse model but also further reveal the potential restriction of PDX tumor mouse model due to the differential expression profile between PDX tumor tissue and clinical samples. The detailed analysis of optimal genes can be seen below.

### 4.1. Differentially Expressed Genes

Among our predicted differentially expressed gene, *EMP1* is at the top rank. In encoding a tumor-associated membrane protein, this gene has been confirmed to be downregulated during the passage and transplantation of breast cancer tumor cells [12,13]. 

The second gene *PARP2* has also been predicted to have differential expression patterns in our study. According to recent publications, this gene has been confirmed to contribute to DNA repair regulation in tumor tissues; this finding is quite common and significant in tumor malignant tissues [49,50]. Studies based on PDX model further confirmed that this gene mediates the chemotherapy resistance of breast cancer and has a differential expression pattern between breast cancer PDX model and corresponding tumor tissue in situ [50,51]. Previous findings demonstrated that during the transplantation processes, the biological process of DNA repair is inhibited, which indicates that *PARP2* might be lowly expressed in PDX model [52].

The third ranked gene *KRT19* has been widely reported to contribute to breast cancer tumorigenesis by its involvement in the organization of myofibers [53,54]. This gene is differentially expressed in PDX model compared with the original tumors not only in breast cancer but also in pancreatic cancer and hepatocellular carcinoma [55,56,57]. The upregulation of this gene helps maintain the structural integrity of epithelial cells [58]. During the transplantation of primary tumor into the mouse model, epithelial cell integrity became unstable in the PDX mouse model; this finding indicates that this gene can be downregulated in PDX model [58]. Interestingly, the homolog of *KRT19* and *KRT5* is also in the optimal feature set. The products of these two genes may form a specific dimer and contribute to specific biological processes as a whole [59]; this observation suggests that this gene can also be differentially expressed in the PDX mouse model.

In encoding a specific membrane glycoprotein, *MUC1* is usually expressed in the epithelial female cancer subtypes (e.g., ovarian cancer and breast cancer) and regarded as the biomarker of cancer cell stemness [60,61,62]. A recent study on the stemness of cancer cells confirmed that during the passage of PDX mouse model, the stemness of cancer cells is gradually shaped by the murine microenvironment [62]; this finding indicates that the expression of *MUC1* can be altered in this progress. Similarly, another gene involved in the stemness maintenance of cancer cells, *PROM1* [55,63], can also have differential expression patterns.

*CXCR4* encodes a specific G protein coupled receptor, which has been widely reported to be expressed on the immature CD34^+^ hematopoietic stem cells [64,65]. Recent studies on breast cancer found that the expression profiles of *CXCR4* are very diverse among the primary tumor of breast cancer, the metastatic tumor tissues, and the PDX tumors filtered by specific murine microenvironment [66,67]. 

In our results, two specific Erb-B2 Receptor Tyrosine Kinase (ERBB)-pathway-associated genes, i.e., *ERBB2* and *ERBB3*, have been predicted as potential distinctive biomarkers. ERBB family signaling pathway has been widely reported to contribute to transcriptional regulation during the tumorigenesis of breast cancer [68]. Further studies on the detailed expression pattern of these two genes in clinical tumor tissues and PDX models confirmed that after the selection of murine tumor microenvironment and PDX model passage, the expression patterns of *ERBB2* and *ERBB3* have been systematically regulated for further adaption to the murine mouse microenvironment [55,69].

Another gene, *ID4*, has also been identified as a breast-cancer-associated gene, which participates in the regulation of basic helix-loop-helix transcription factors [70,71]. Although no direct evidence is found on the differential expression pattern in paired PDX mouse model and tumor tissues in situ, the expression of creatine kinase is altered due to the microenvironment of PDX mouse model [72,73]. As the negative regulator of creatine kinase, the expression pattern of *ID4* may also be changed in a different microenvironment.

A recent study on single-cell transcriptome of PDX mouse model reported a series of genes with differential expression patterns in the PDX mouse model compared with the original tumors [13]; this series included *PTEN*, *NTRK2*, *PGR*, and *TP53*, which are identified in this study as the differentially expressed genes.

### 4.2. Rules of Quantitative Expression Level Requirements

As we have mentioned above, we detected seven specific rules to distinguish primary tumor tissues and PDX tumor tissues based on the expression levels of 14 genes. In the seven rules, the first six provided criteria for classifying a tumor cell into the original human tumor, which also represents the primary tumor-specific gene expression patterns (see Table 2). Among the 14 genes, seven genes, i.e., *KRT19*, *EMP1*, *CXCR4*, *CD44*, *PTEN*, *PARP2*, and *PLCB4*, were required to have high expression levels in the original tumor cells, whereas others should have low expression levels. As we discussed above, some of these genes, such as *KRT19*, *EMP1*, and *PARP2*, have evidence from previous studies, which indicate their higher expression levels in the original tumors compared with PDX tumor cells [12,13,52,58]. 

**Figure 3:**
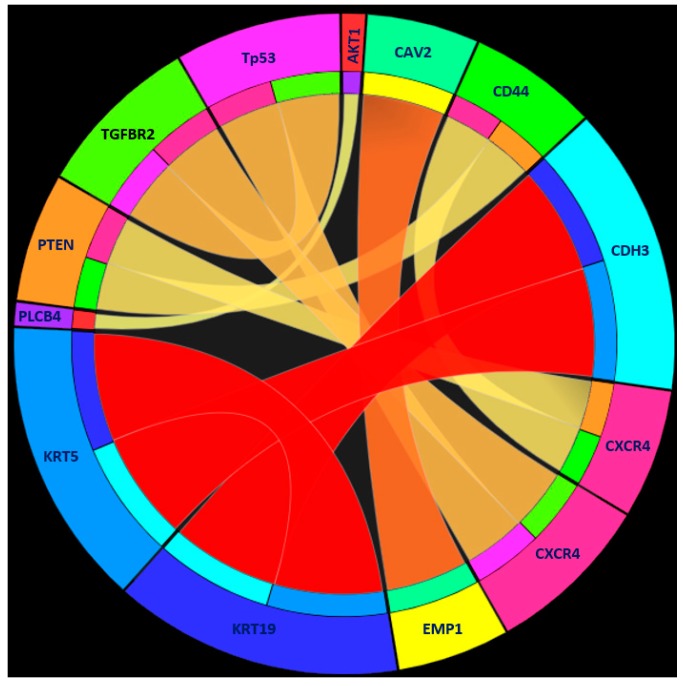
Rule networks for the seven detected rules generated by Ciruvis [74]. For the purpose of visualization, Ciruvis assigned specific colors for genes and placed genes into a circle. Meanwhile, Ciruvis indicated the strength of interaction among genes by using the degree of red color on the interactions. The darker the red color is, the stronger the interaction among genes.

Further, to facilitate our understanding of the seven rules, we visualized the seven rules by using Ciruvis, a web-based tool for rule networks and interaction detection using rule-based classifiers, as suggested in the literature [74]. The result is shown in Figure 3, from which we found that, strong interactive relationships involved genes of *KRT19*, *KRT5* and *CDH3* (interactions of dark red color). Thus, we provided full analysis on the interactions among *KRT19*, *KRT5* and *CDH3*.

First, the dimerizing of Krt5 with Krt14 has been confirmed to be involved in the regulation of EMT in tumorigenesis [75,76,77]. Further studies on the protein–protein interactions confirmed that this heterodimer could also interact with another component of cytokeratins, Krt6b [78]. As a functional mediator, Krt6b connects Krt5 with Krt19 through protein–protein interaction [79]. Krt6b shows negatively correlated expression pattern with Krt19 during tumorigenesis [80]. Therefore, the strong interaction between *KRT19* and *KRT5* has been indicated by previous experiments. 

Second, the Ciruvis figure also show strong correlations between *KRT5* and *CDH3*, which encodes a member of the cadherin superfamily. A previous study in 2010 [81] reported that *BRCA1* repressed the expression of various cytokeratins including Krt5. It is also known that BRCA1 product manipulate the expression pattern of both *KRT5* and *CDH3* [81], suggesting the correlation and co-regulation of the expressions of these two genes. 

Third, the correlation of *KRT19* and *CDH3* is suggested by the previous studies which reported the participation of these two genes in the essential proliferation and metastasis associated pathway MAPK/ERK cascade in tumorigenesis [82,83]. Furthermore, *KRT19* shows co-expression pattern with various *CDH3* associated genes like *CDH1* [84], *EGFR* [85] and *CTNNB1* [54] in certain pathological conditions, indicating that there may be potential biological relevance between *KRT19* and *CDH3*. 

In summary, these findings strongly support the correlations of both functions and expression patterns among the three genes *KRT19*, *KRT5* and *CDH3*, which are all related to EMT associated pathological processes in human tumors, but not in PDX mouse model.

## 5. Conclusions

Through a new computational method, we identified the differentially expressed genes and quantitative rules that can classify the PDX tumor and the original tumor with high accuracies. Multiple well-known oncogenes and tumor suppressors are differentially expressed; this finding indicates the risk of using those genes as therapeutic targets in breast cancer through PDX mouse model. Therefore, our study not only provides a functional tool to distinguish the primary tumor from PDX tumor by gene expression levels but also reveals the detailed gene expression alterations and shaping characteristics in murine microenvironment. In this work, we employed SVM, RF, dagging and rough set classifiers due to the small samples problem. Some of the latest classifiers, such as ensemble classifier [86], will be tested in future work.

## Figures and Tables

**Table 1:** Comparison on performance of different models. The RF model with top 32 features achieved the highest MCC (0.777). RF: random forest; SVM: supporter vector machine; MCFS: Monte Carlo feature selection.

Model	Feature No.	MCC	Sensitivity	Specificity	Accuracy
RF	32	**0.777**	0.996	0.672	0.929
RF	57 (MCFS cutoff)	0.695	0.995	0.598	0.905
Rough Set	57 (MCFS cutoff)	0.665	0.950	0.680	0.893
SVM	41	0.695	0.995	0.563	0.904
Dagging	58	0.599	0.996	0.436	0.878

**Table 2:** Seven rules quantitatively defining the criteria for classification. These rules were produced by the MCFS software package. If a tumor cell satisfies the first six criteria, it would be classified into Human tumor; otherwise, it would be classified into PDX tumor.

Rules	Criteria	Classification
Rule 1	KRT19 ≥ 1.939224	Human tumor
	KRT5 ≤ 0.148786	
	CDH3 ≤ 0.868794	
Rule 2	EMP1 ≥ 4.237572	Human tumor
	CAV2 ≤ 1.610886	
Rule 3	TP53 ≤ 0.291193	Human tumor
	CXCR4 ≥ 4.367387	
	TGFBR2 ≤ 1.868461	
Rule 4	CXCR4 ≤ −2.474571	Human tumor
	CD44 ≥ 0.086944	
	PTEN ≥ 0.143515	
	VIM ≤ 0.647694	
Rule 5	PARP2 ≥ 3.111536	Human tumor
Rule 6	PLCB4 ≥ 3.744729	Human tumor
	AKT1 ≤ −0.070679	
Rule 7	Other conditions	PDX tumor

PDX: Patient-derived tumor xenograft.

**Table 3:** Thirty-two differentially expressed genes identified as optimal features.

HUGO Symbol	HUGO Name	*RI*
*EMP1*	epithelial membrane protein 1	0.16895404
*PARP2*	poly(ADP-ribose) polymerase 2	0.15058246
*KRT19*	keratin 19	0.12158414
*MUC1*	mucin 1, cell surface associated	0.11115772
*CXCR4*	C-X-C motif chemokine receptor 4	0.07917199
*PROM1*	prominin 1	0.06480689
*ERBB2*	erb-b2 receptor tyrosine kinase 2	0.048957534
*ERBB3*	erb-b2 receptor tyrosine kinase 3	0.04209958
*KRT5*	keratin 5	0.037512265
*ID4*	inhibitor of DNA binding 4, HLH protein	0.03389286
*PTEN*	phosphatase and tensin homolog	0.029668033
*NTRK2*	neurotrophic receptor tyrosine kinase 2	0.022596486
*PGR*	progesterone receptor	0.020494139
*TP53*	tumor protein p53	0.019557578
*CDH3*	cadherin 3	0.01846532
*BMI1*	BMI1 proto-oncogene, polycomb ring finger	0.013900218
*TGFBR2*	transforming growth factor beta receptor 2	0.013375987
*CCNB1*	cyclin B1	0.013296658
*PLCB4*	phospholipase C beta 4	0.013219586
*CLDN4*	claudin 4	0.013182897
*CXCL12*	C-X-C motif chemokine ligand 12	0.010324035
*EGFR*	epidermal growth factor receptor	0.010273729
*CD44*	CD44 molecule (Indian blood group)	0.009676576
*LGR5*	leucine rich repeat containing G protein-coupled receptor 5	0.008659011
*NOTCH4*	notch 4	0.007799821
*BCL2*	BCL2, apoptosis regulator	0.007518955
*CAV2*	caveolin 2	0.007474113
*VEGFC*	vascular endothelial growth factor C	0.006789302
*TGFBR1*	transforming growth factor beta receptor 1	0.006149265
*VIM*	vimentin	0.005953075
*TGFB2*	transforming growth factor beta 2	0.005226418
*KRT8*	keratin 8	0.00506866

HUGO: Human gene nomenclature; *RI*: relative importance.

## Supplementary Materials

The following are available online at http://www.mdpi.com/2073-4425/9/3/155/s1. Supplementary Material I: Incremental forward selection by using Random Forest, Supplementary Material II: The significant KEGG and GO enrichment results of 32 identified genes with FDR < 0.05, Supplementary Material III: Details of rough-set based model.
