# Supplementary material for: Identification of Differentially Expressed Genes between Original Breast Cancer and Xenograft Using Machine Learning Algorithms
Source: Genes (Basel). 2018 Mar 12;9(3):155. doi: 10.3390/genes9030155 (PMC5867876; doi:10.3390/genes9030155)
Supplement: Supplementary file 1 [file genes-09-00155-s001.zip › Supplementary Material III.docx]

**Details of rough-set based model**

***A. The seven rules provided by using RIPPER algorithm***

**Rule 1:** (GENE_KRT19 >= 1.939224) and (GENE_KRT5 <= 0.148786) and (GENE_CDH3 <= 0.868794) => class=HUMAN TUMOR (75.0/5.0)

**Rule 2:** (GENE_EMPDX TUMOR >= 4.237572) and (GENE_CAV2 <= 1.610886) => class=HUMAN TUMOR (34.0/1.0)

**Rule 3:** (GENE_TP53 <= 0.291193) and (GENE_CXCR4 >= 4.367387) and (GENE_TGFBR2 <= 1.868461) => class=HUMAN TUMOR (24.0/6.0)

**Rule 4:** (GENE_CXCR4 <= -2.474571) and (GENE_CD44 >= 0.086944) and (GENE_PTEN >= 0.143515) and (GENE_VIM <= 0.647694) => class=HUMAN TUMOR (20.0/4.0)

**Rule 5:** (GENE_PARHUMAN TUMOR >= 3.111536) => class=HUMAN TUMOR (8.0/1.0)

**Rule 6:** (GENE_PLCB4 >= 3.744729) and (GENE_AKT1 <= -0.070679) => class=HUMAN TUMOR (6.0/1.0)

**Rule 7:** class=PDX TUMOR (664.0/25.0)

The number of rules, support values, coverage and ranking of the rules are shown above. The first number in the bracket (highlighted in yellow color) is the number of instances that are covered by the rule and second number is the number of instances that are misclassified. Please see the description of the RIPPER for how it generates rules in the section 2.4.

***B. Model Performance***

##### Cross Validation Result (10 folds repeated 3 times) #####

Accuracy = 0.8937

True Positive Rate

HUMAN TUMOR: 0.6800

PDX TUMOR: 0.9502

False Positive Rate

HUMAN TUMOR: 0.0497

PDX TUMOR: 0.3199
